# Supplementary material for: Antifungal Activity against Botrytis cinerea of 2,6-Dimethoxy-4-(phenylimino)cyclohexa-2,5-dienone Derivatives
Source: Molecules. 2019 Feb 15;24(4):706. doi: 10.3390/molecules24040706 (PMC6412631; doi:10.3390/molecules24040706)
Supplement: Supplementary file 1 [file molecules-24-00706-s001.pdf]

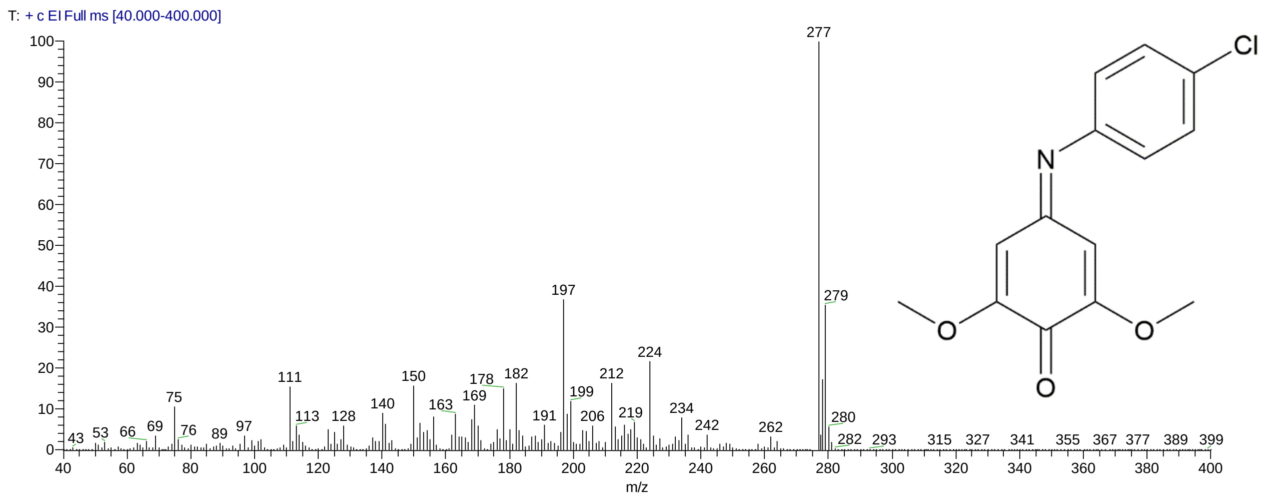

Figure S1. Mass spectrum of compound 3a.

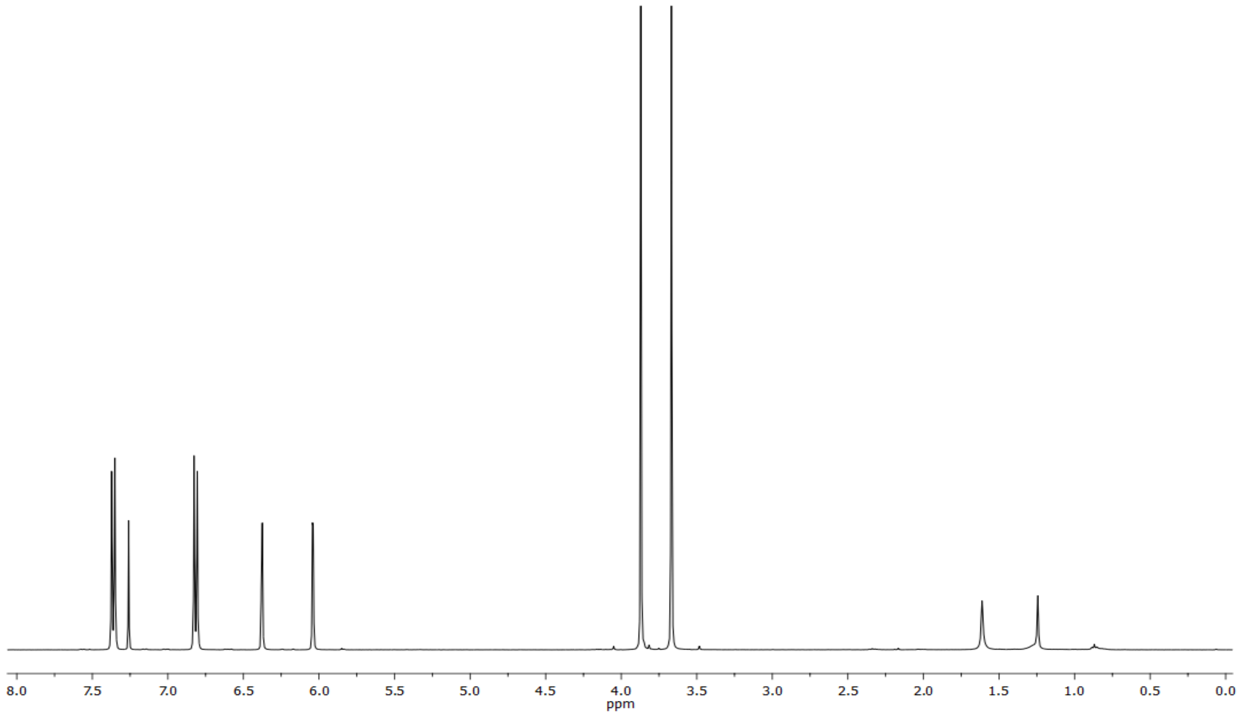

Figure S2. ^{1}H-NMR spectrum of compound 3a.

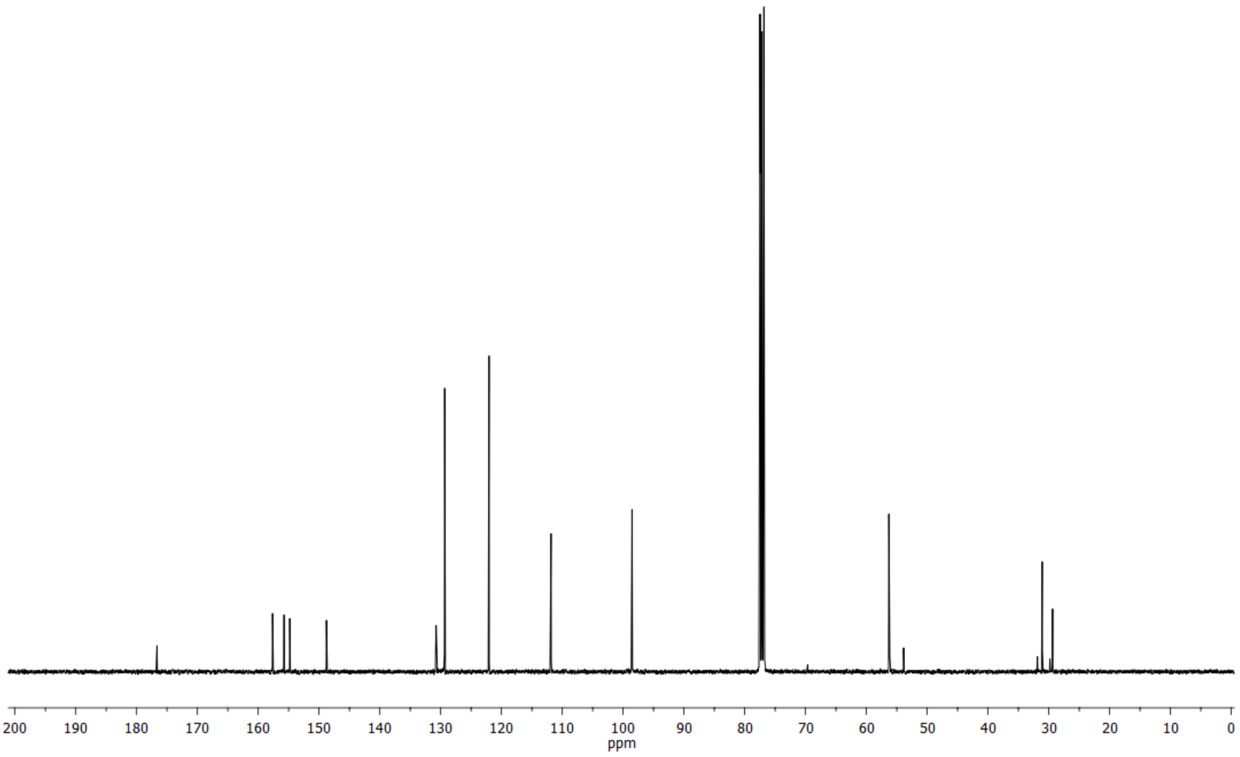

Figure S3. ^{13}C-NMR spectrum of compound 3a.

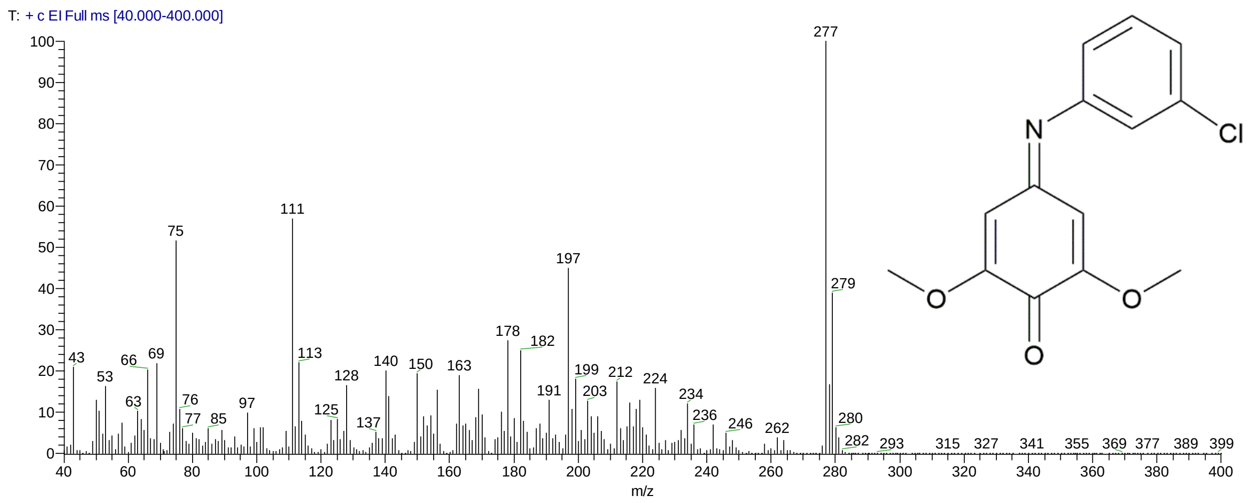

Figure S4. Mass spectrum of compound 3b.

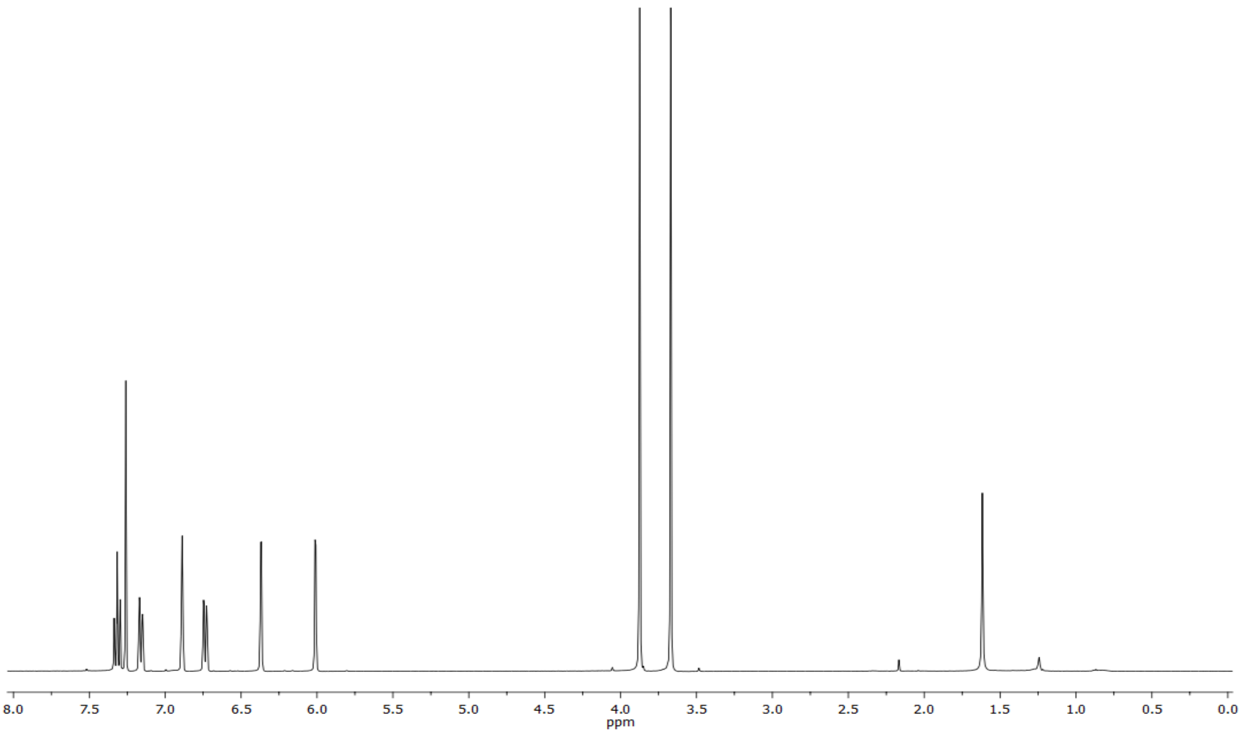

Figure S5. ^{1}H-NMR spectrum of compound 3b.

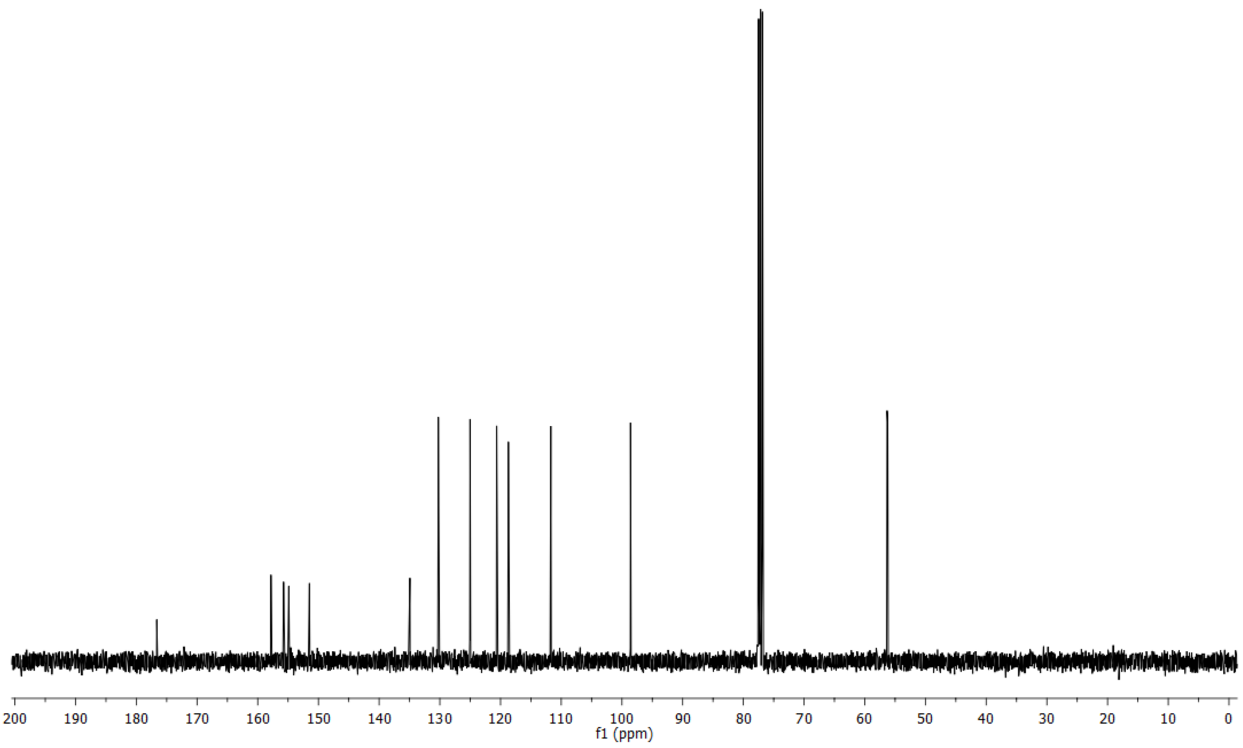

Figure S6. ^{13}C-NMR spectrum of compound 3b.

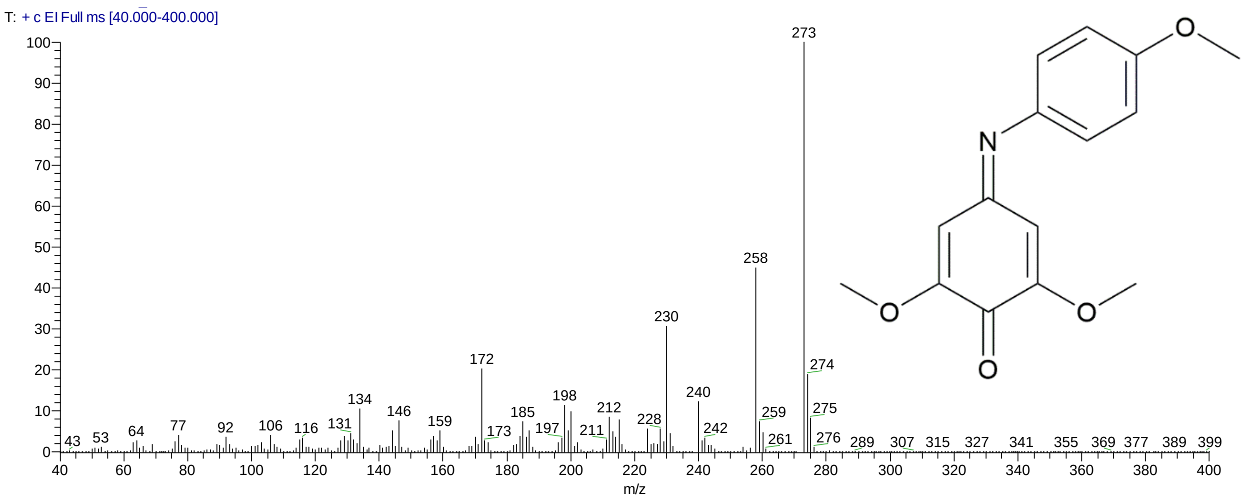

Figure S7. Mass spectrum of compound 3c.

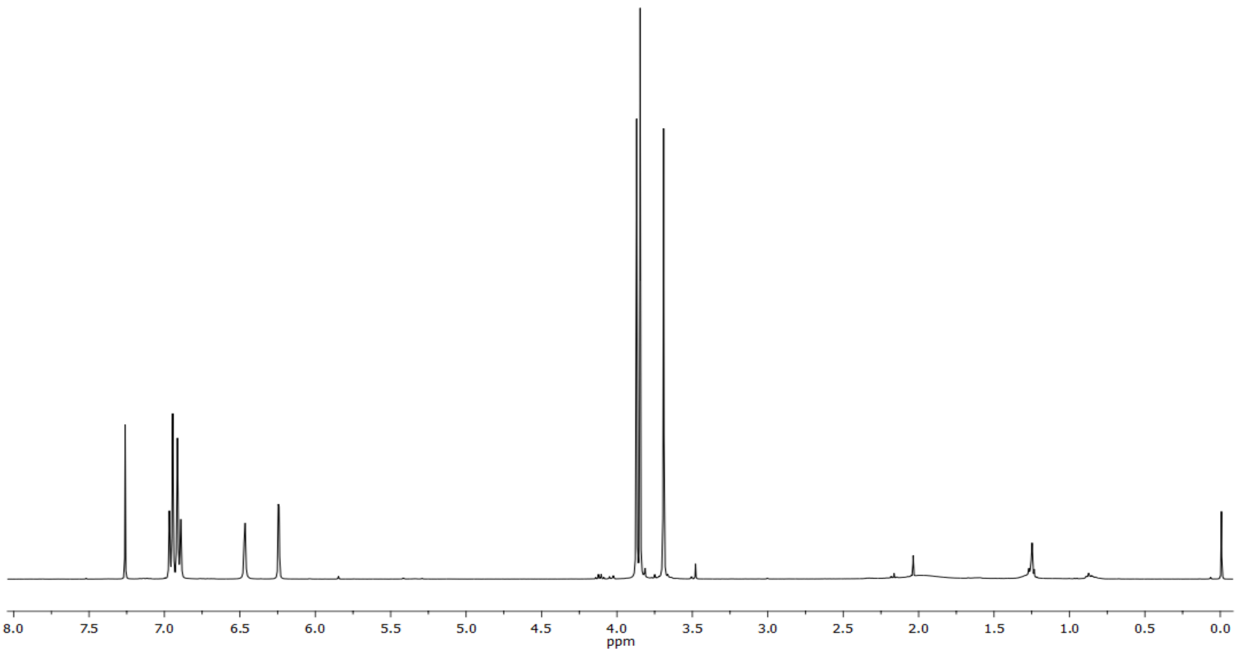

Figure S8. ^{1}H-NMR spectrum of compound 3c.

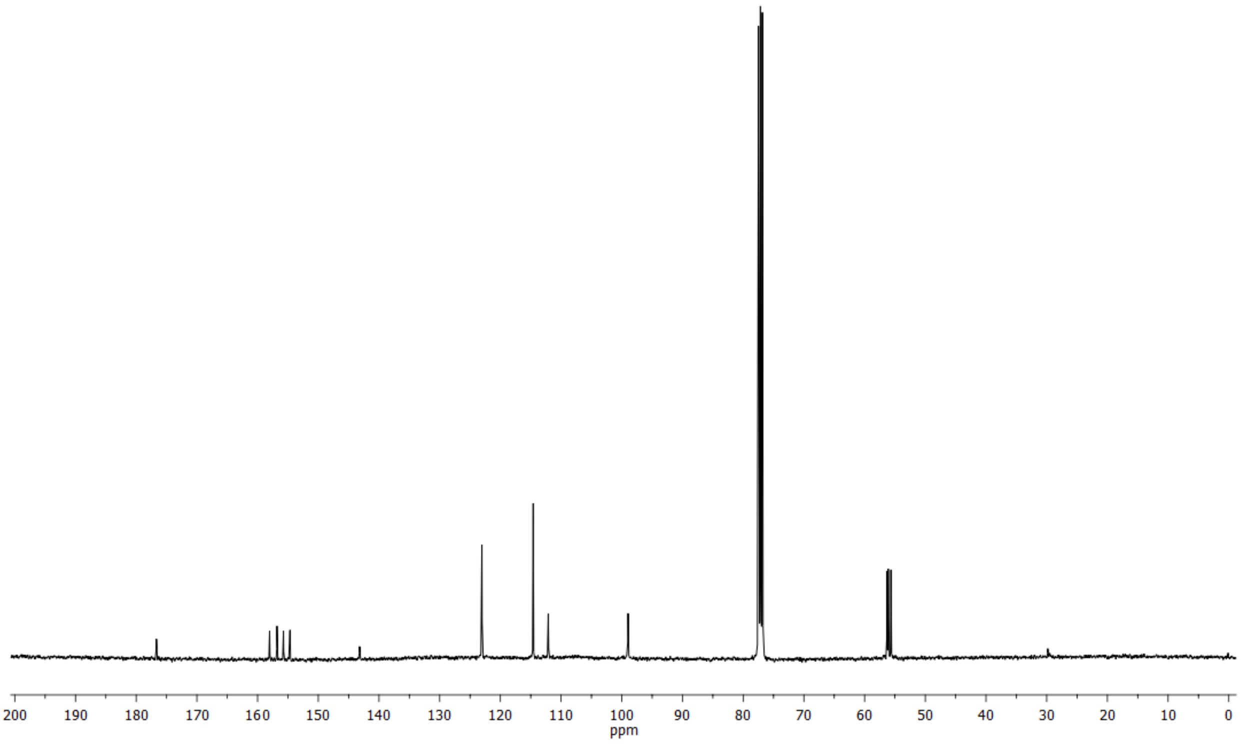

Figure S9. ^{13}C-NMR spectrum of compound 3c.

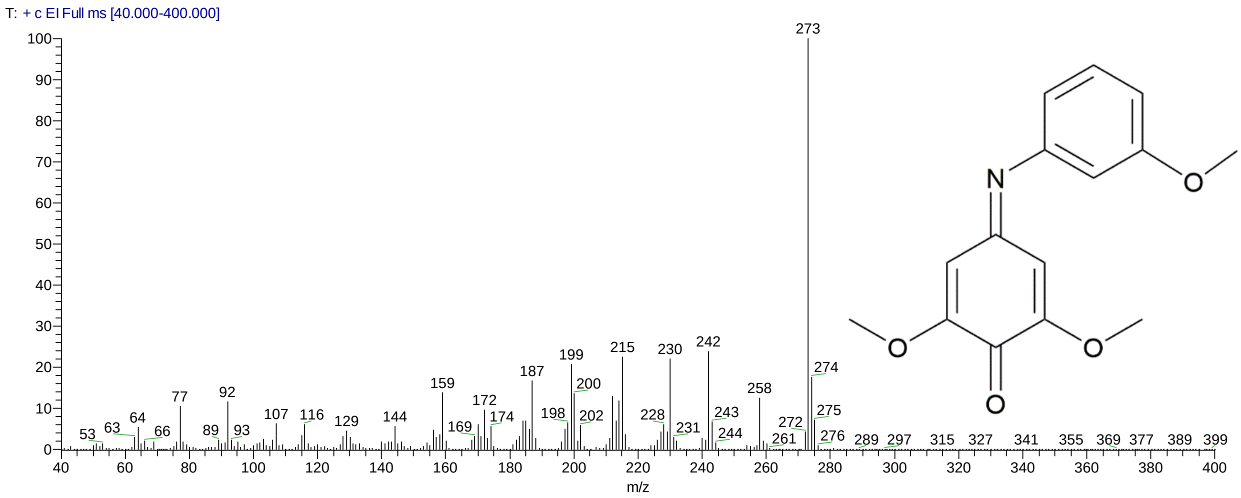

Figure S10. Mass spectrum of compound 3d.

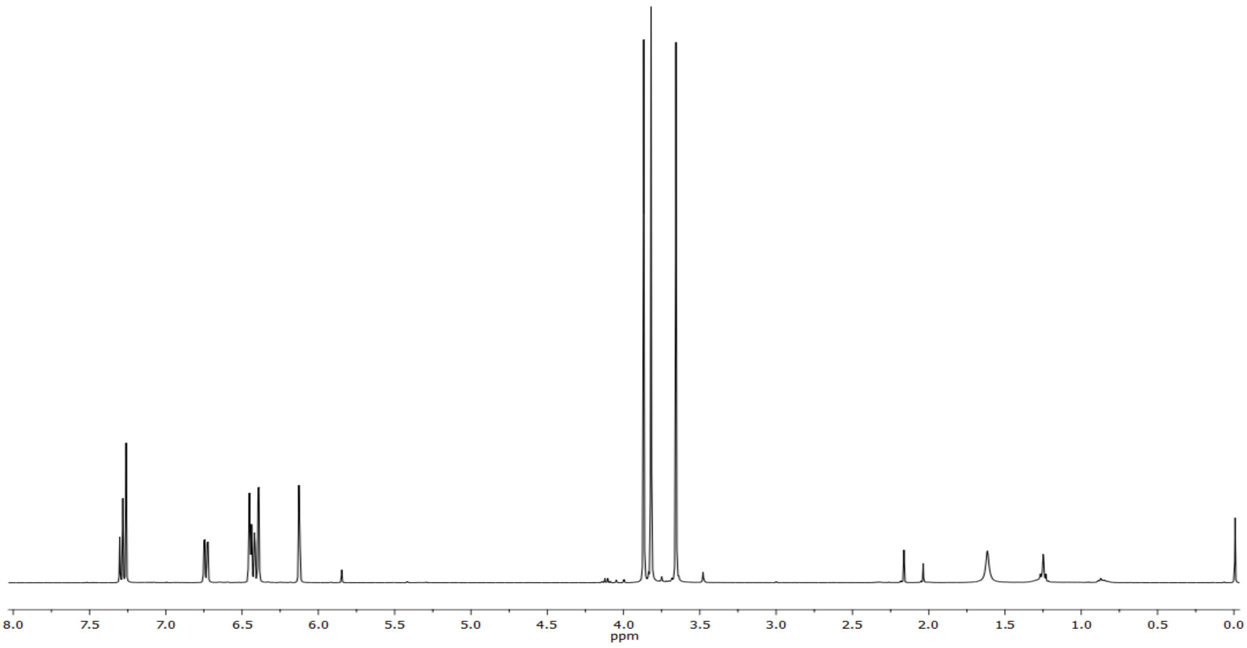

Figure S11. ^{1}H-NMR spectrum of compound 3d.

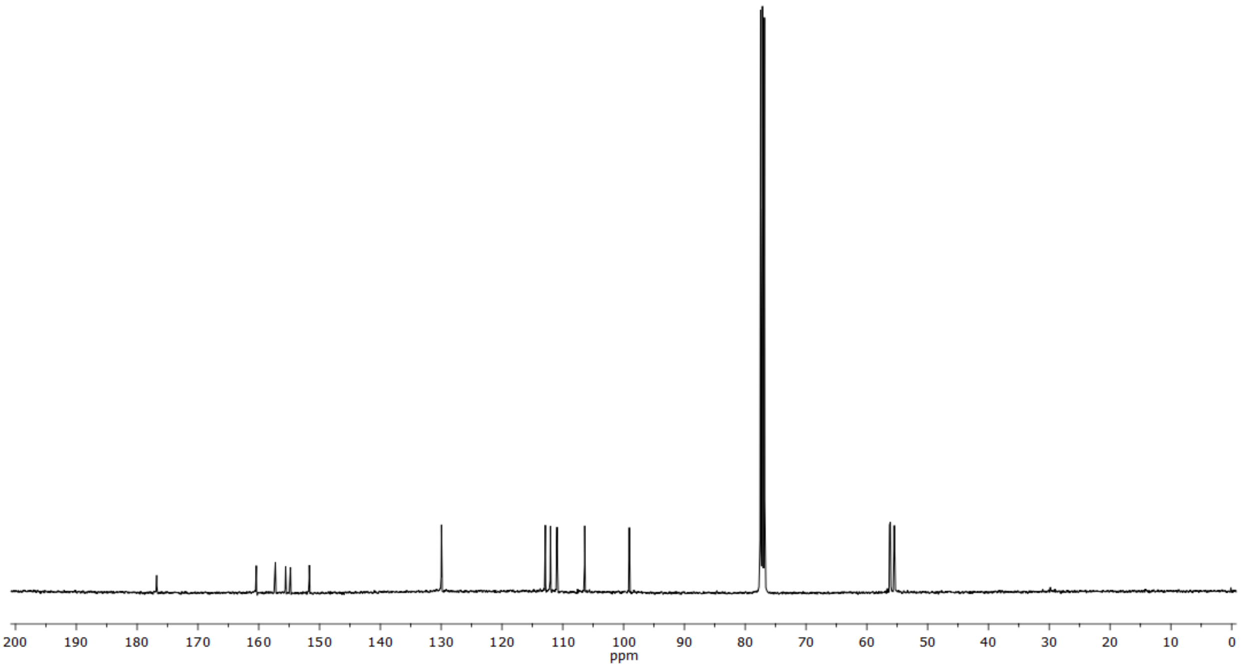

Figure S12. ^{13}C-NMR spectrum of compound 3d.

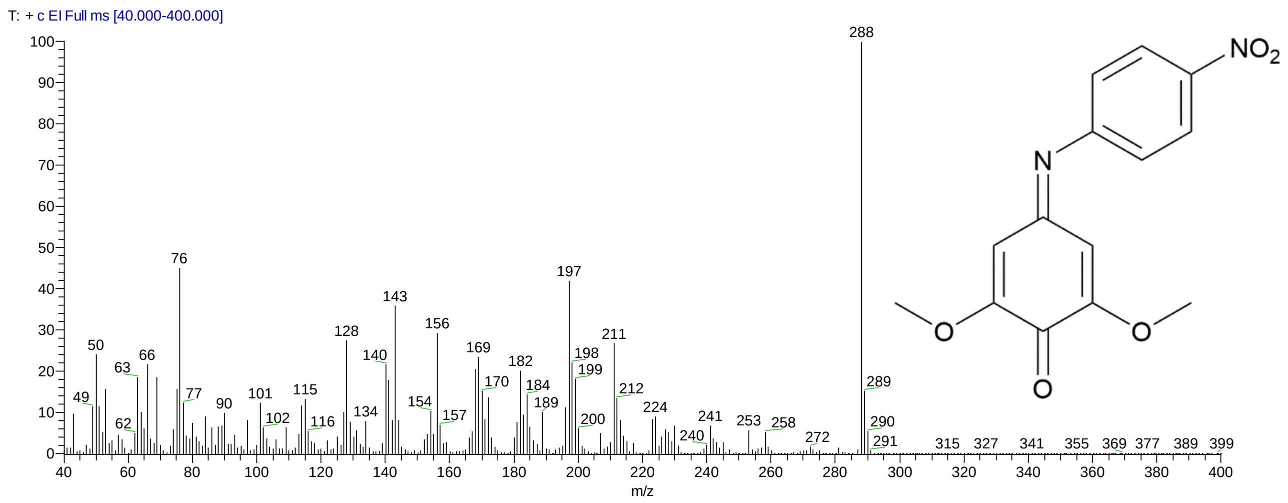

Figure S13. Mass spectrum of compound 3e.

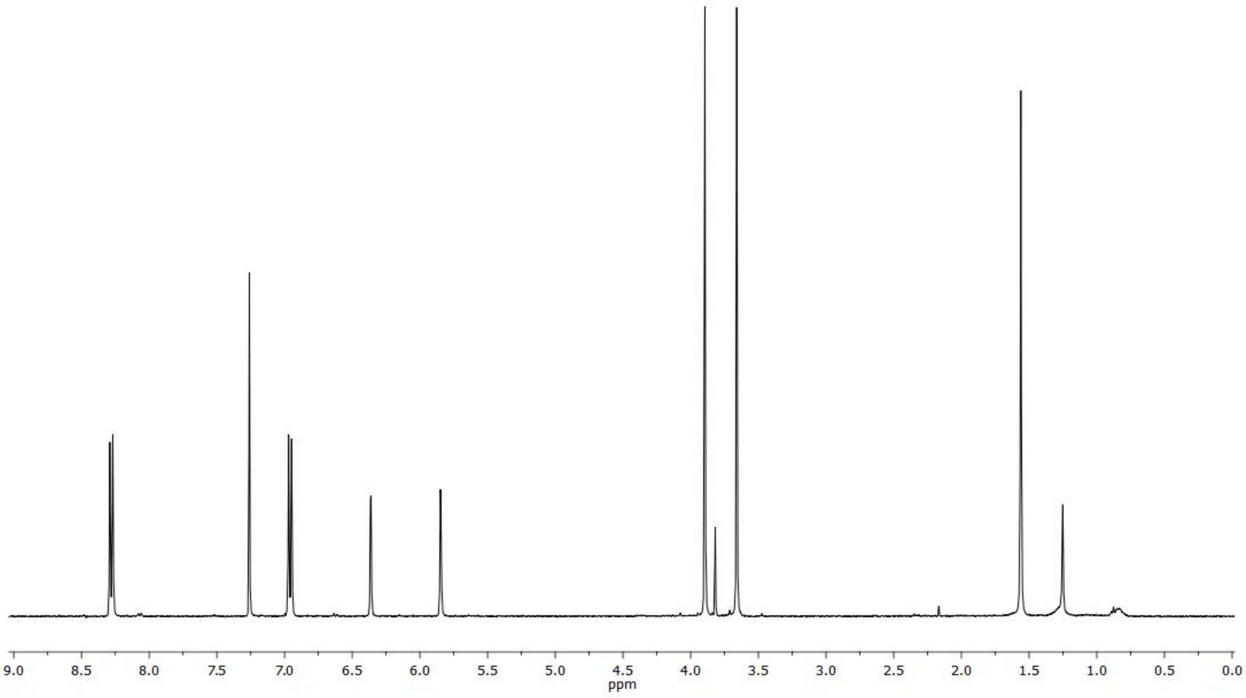

Figure S14. ^{1}H-NMR spectrum of compound 3e.

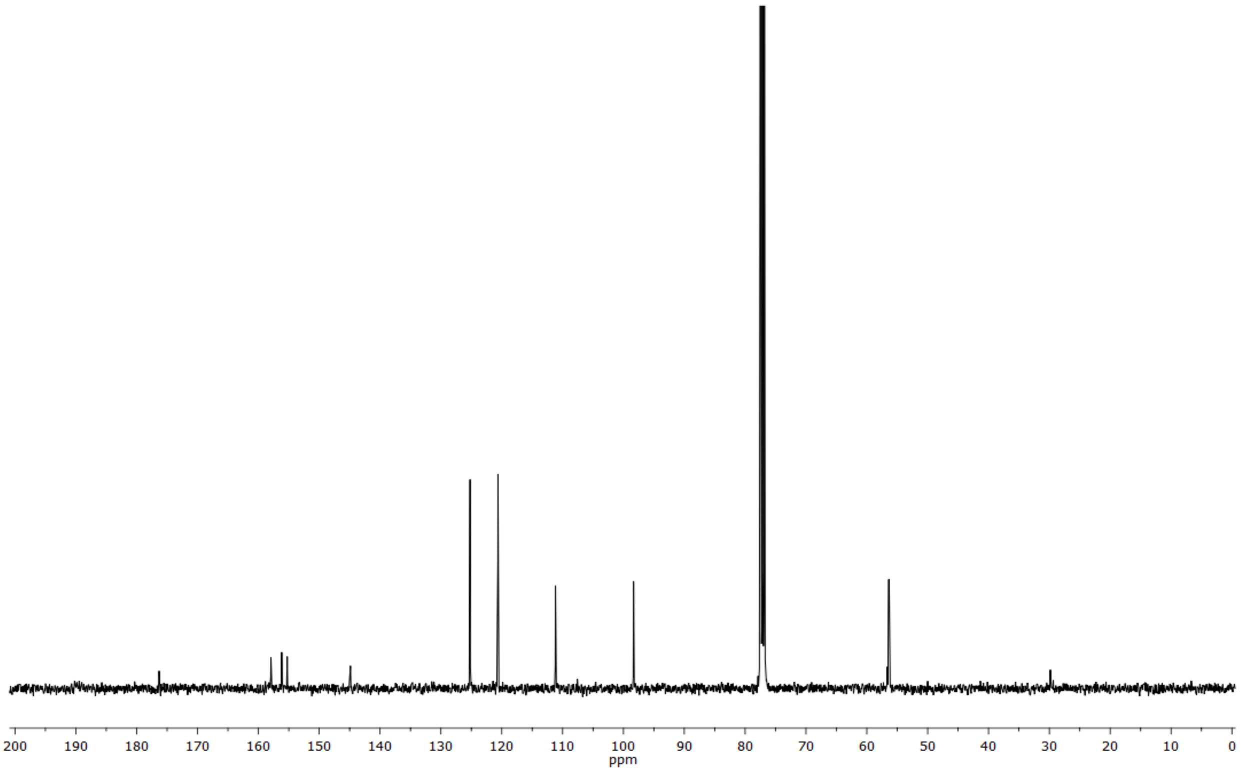

Figure S15. ^{13}C-NMR spectrum of compound 3e.

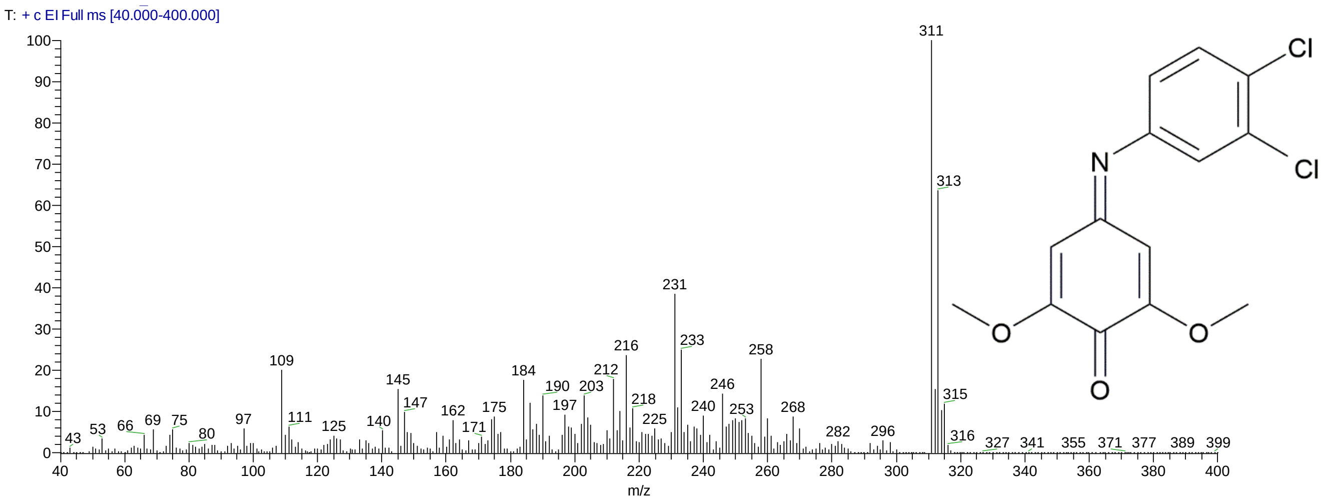

Figure S16. Mass spectrum of compound 3f.

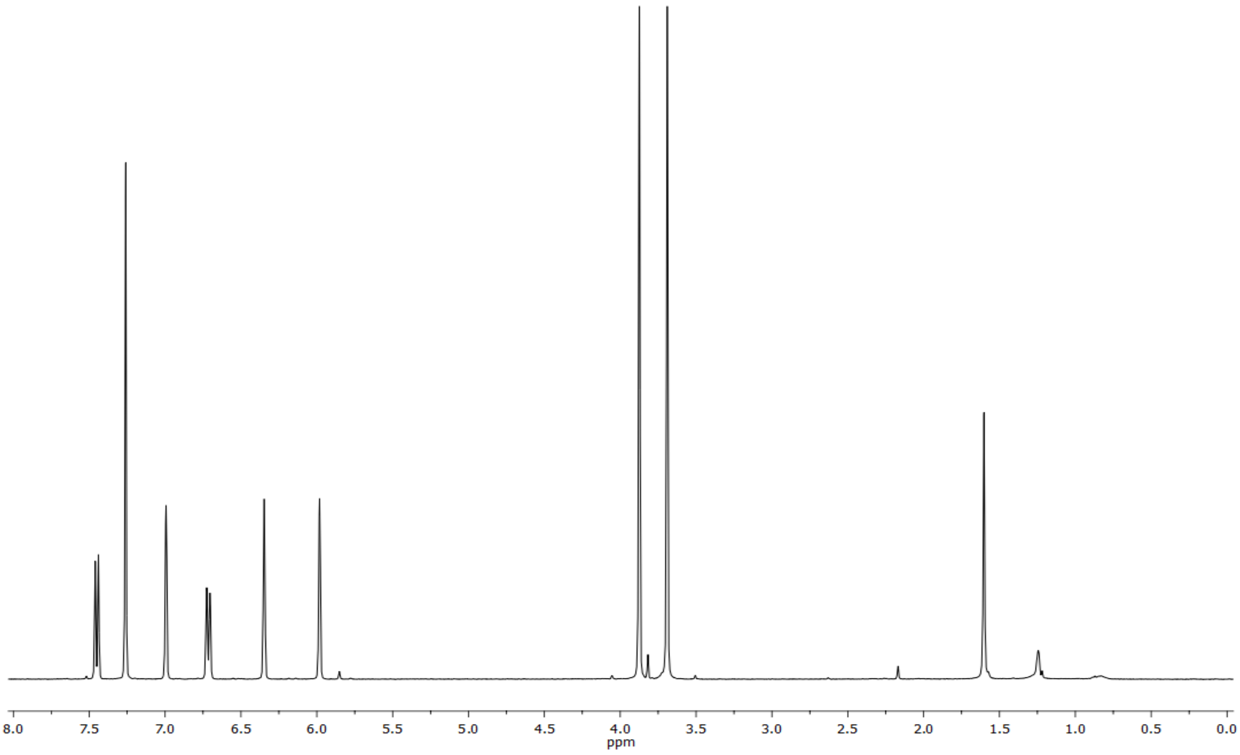

Figure S17. ^{1}H-NMR spectrum of compound 3f.

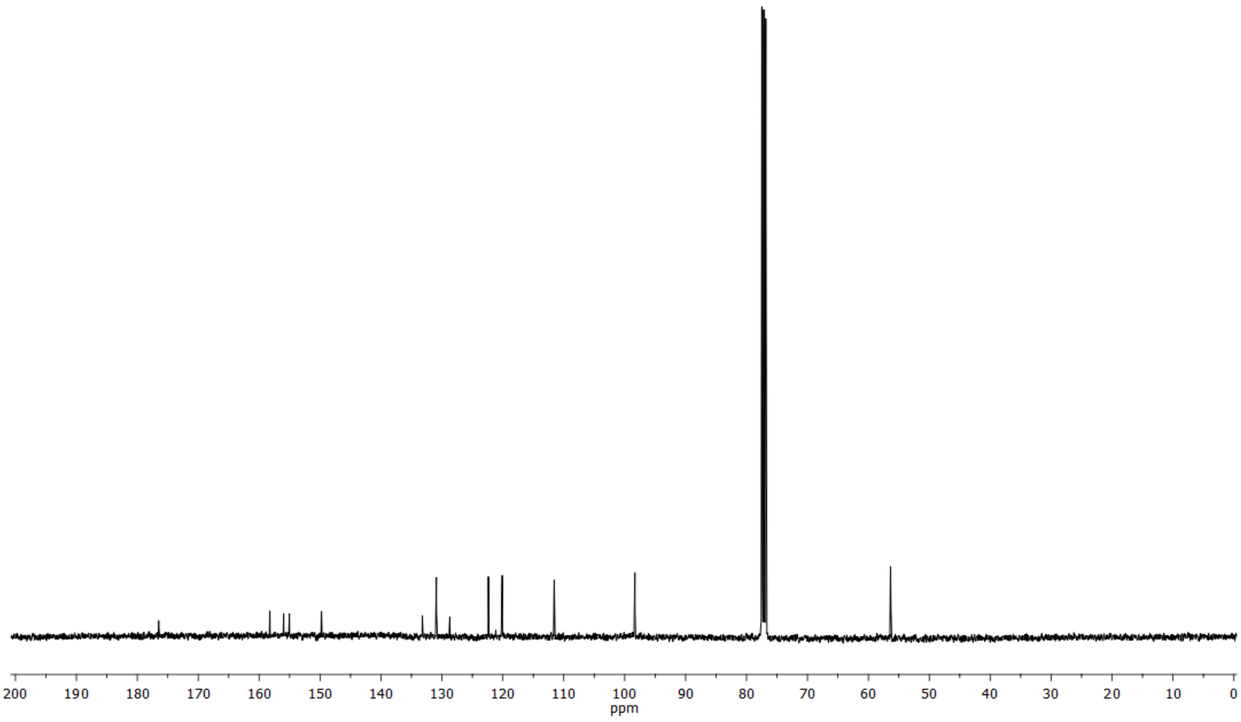

Figure S18. ^{13}C-NMR spectrum of compound 3f.

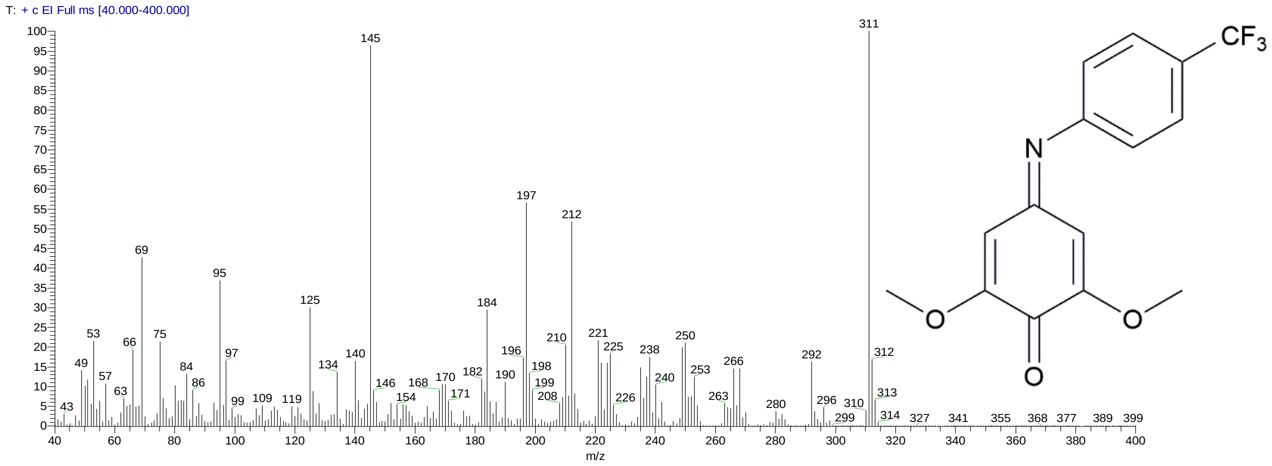

Figure S19. Mass spectrum of compound 3g.

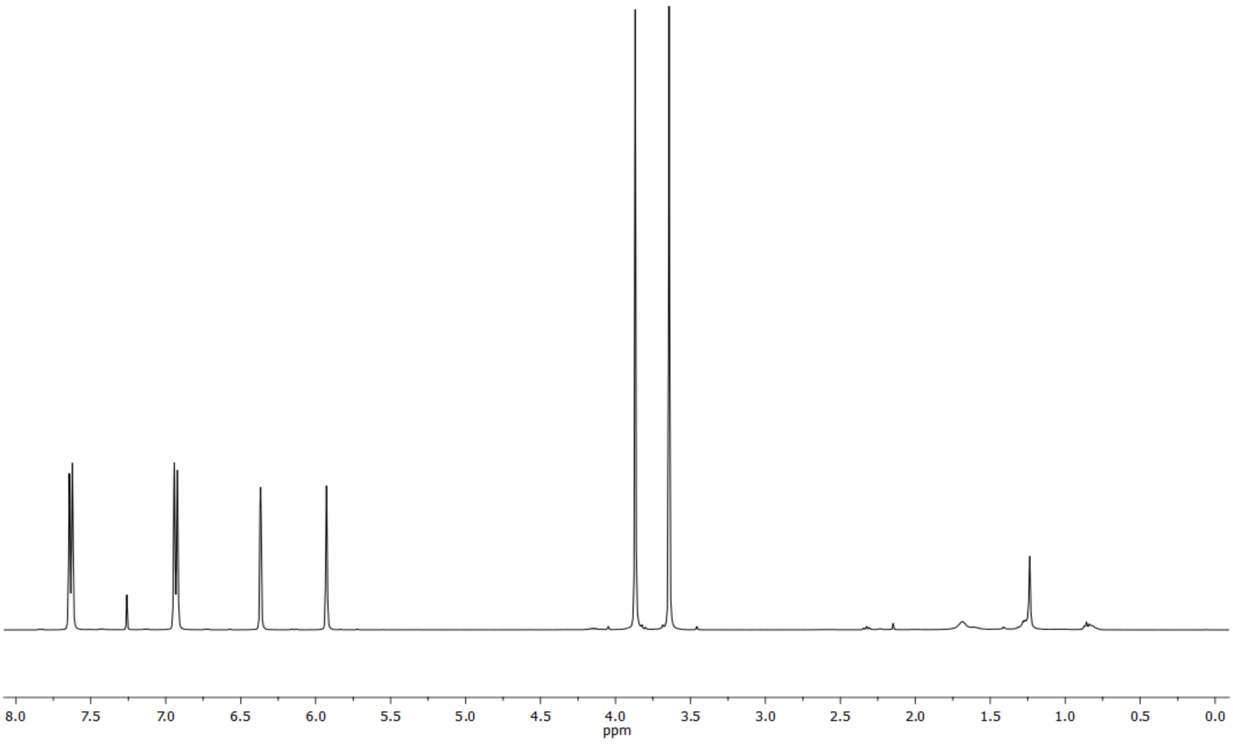

Figure S20. ¹H-NMR spectrum of compound 3g.

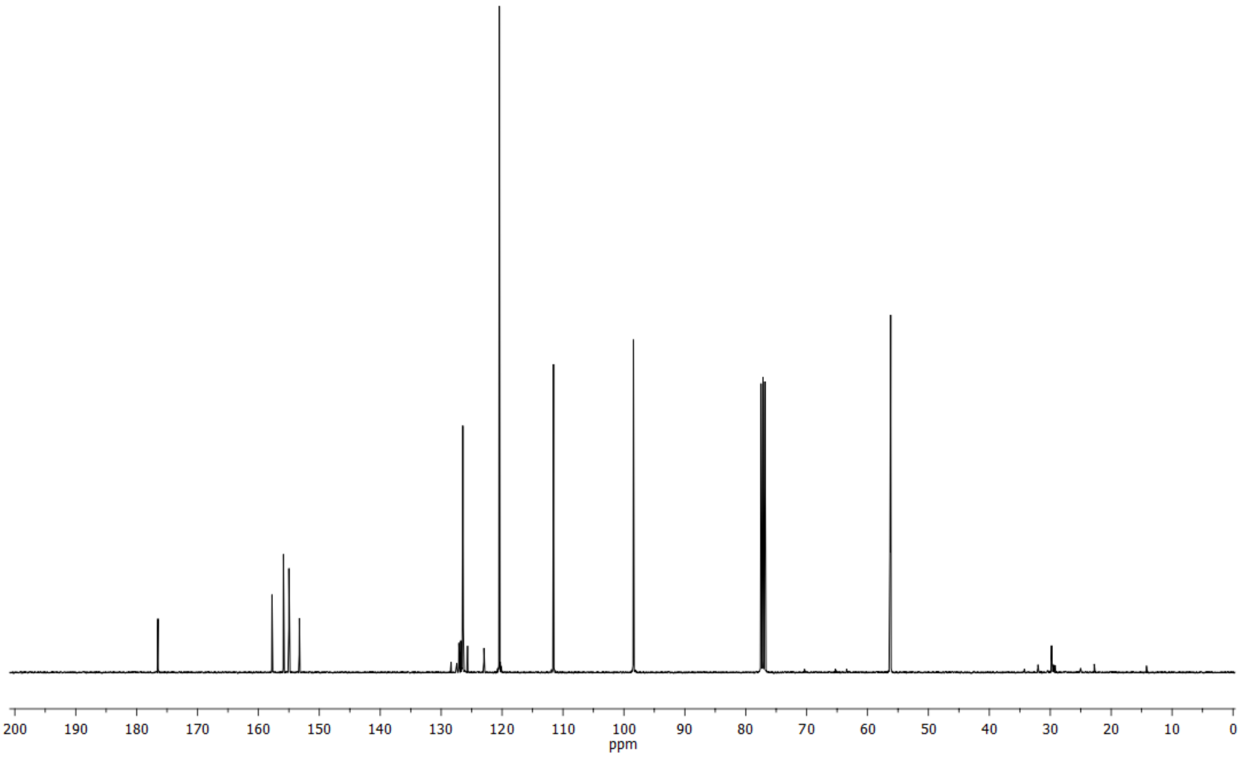

Figure S21. ¹³C-NMR spectrum of compound 3g.

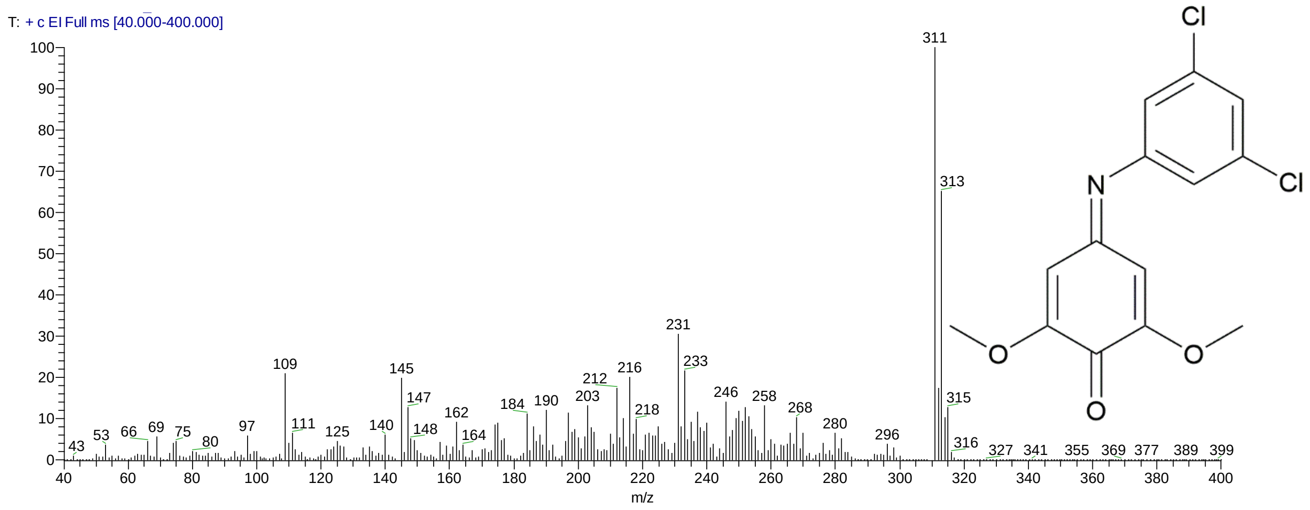

Figure S22. Mass spectrum of compound 3h.

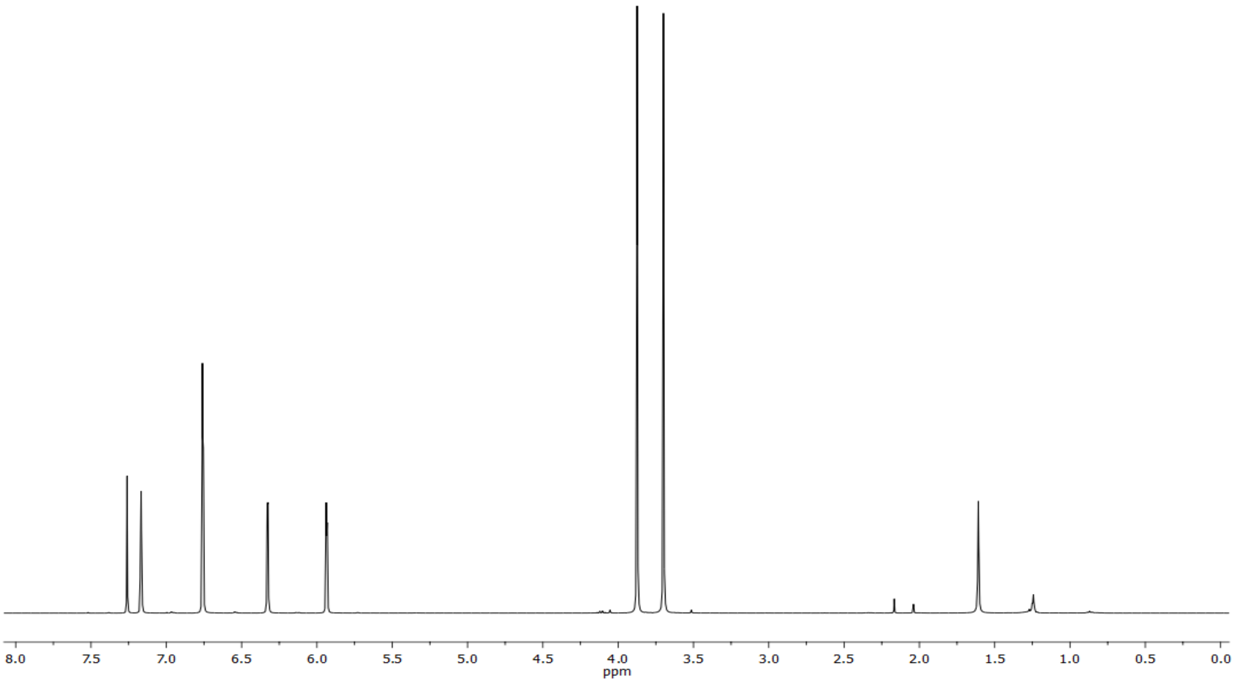

Figure S23. ^{1}H-NMR spectrum of compound 3h.

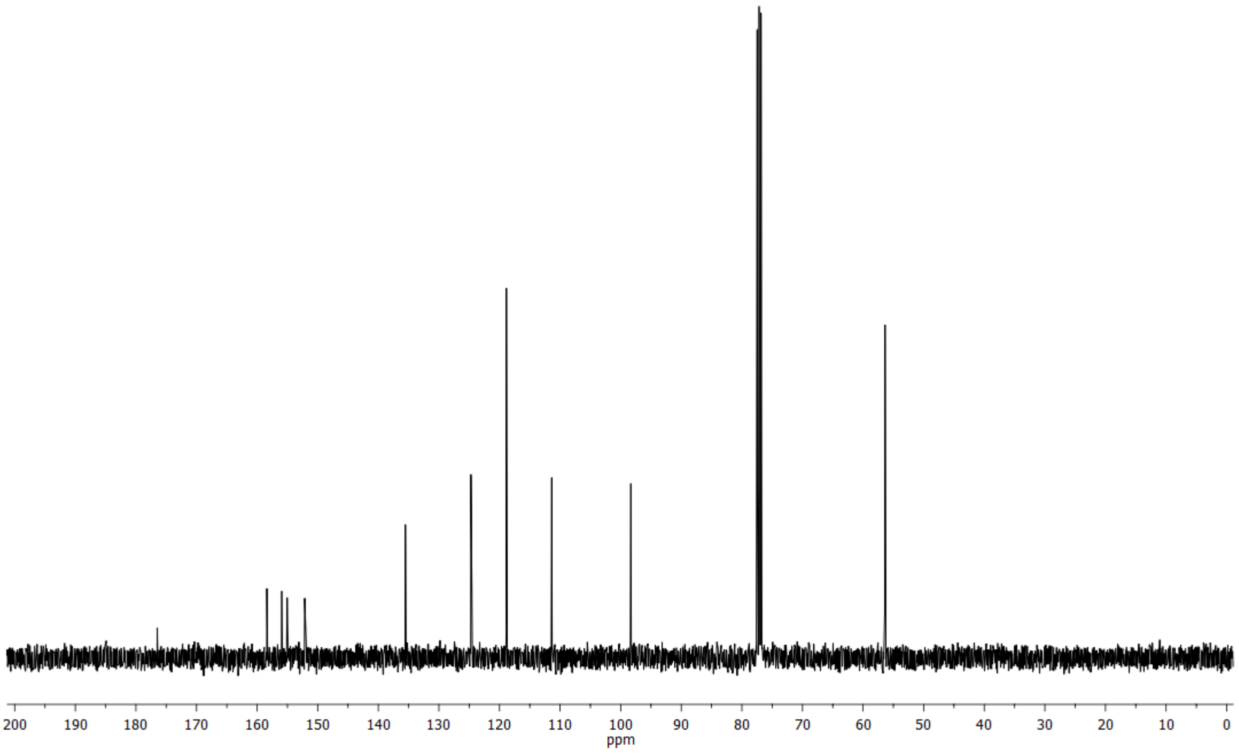

Figure S24. ^{13}C-NMR spectrum of compound 3h.

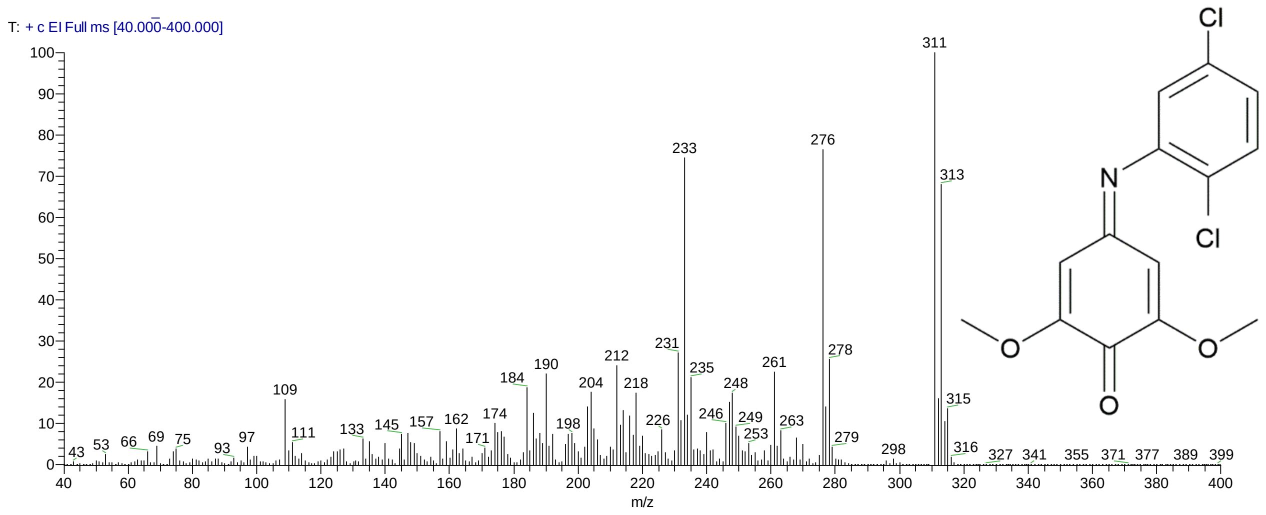

Figure S25. Mass spectrum of compound 3i.

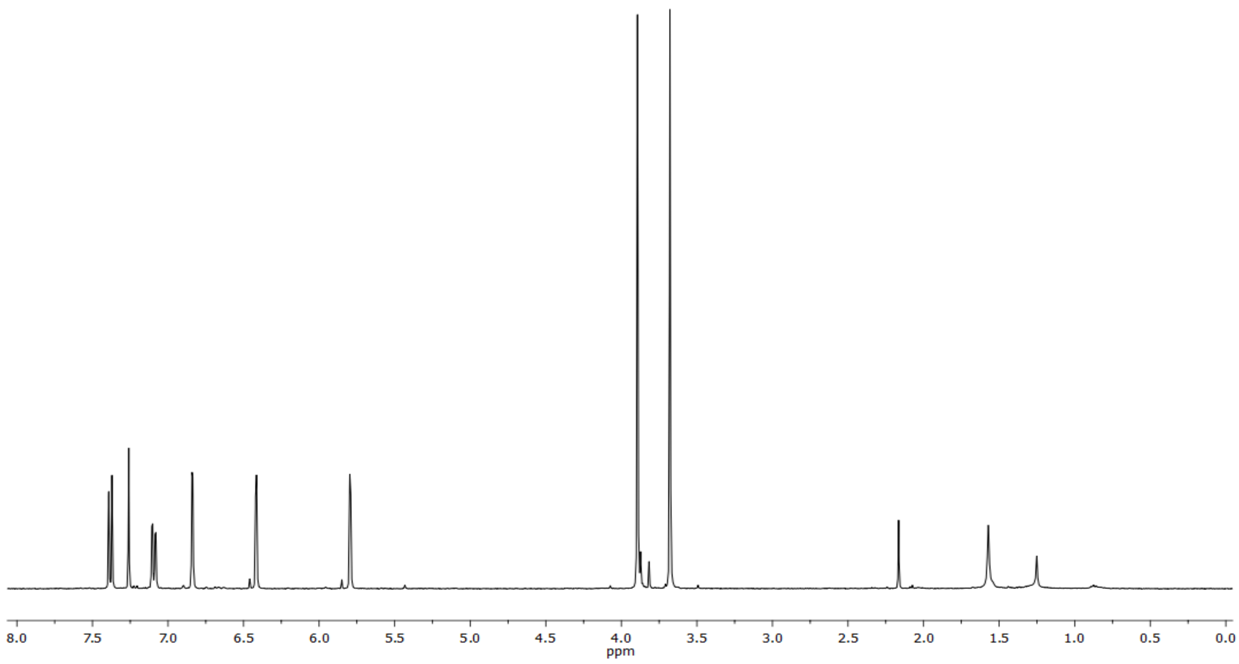

Figure S26. ¹H-NMR spectrum of compound 3i.

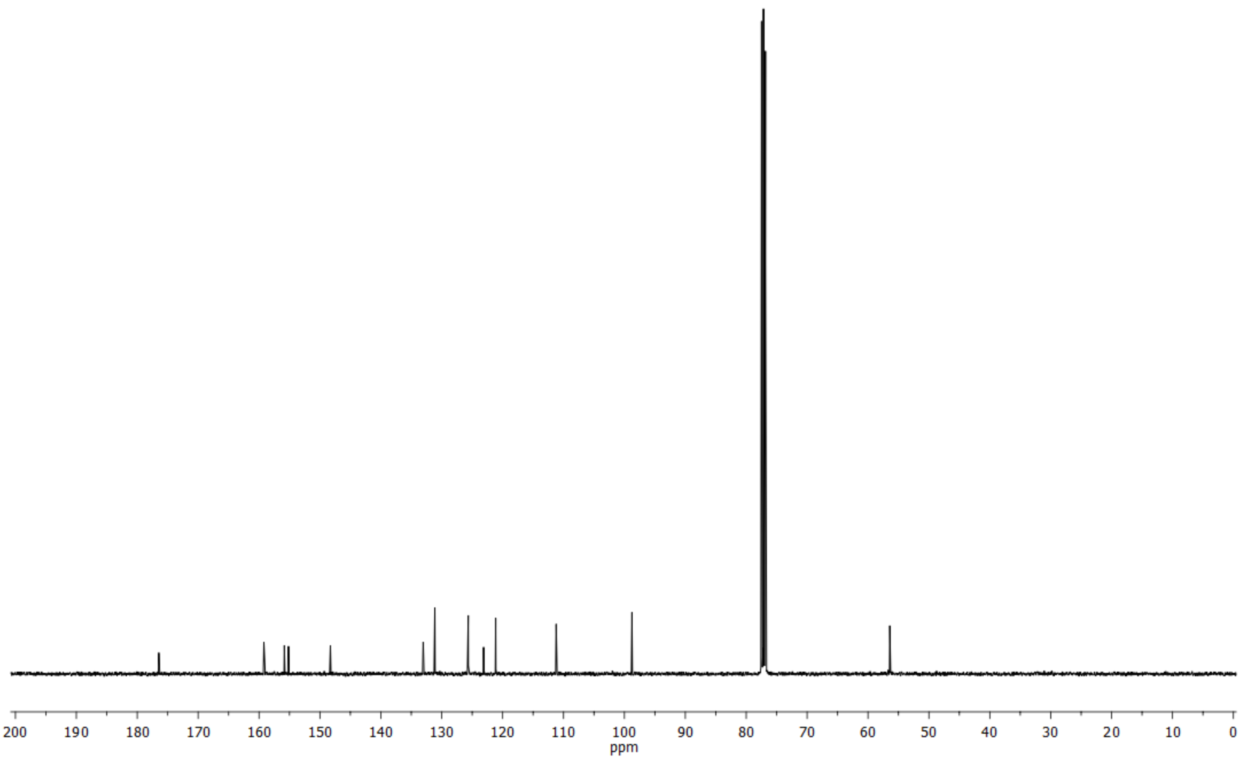

Figure S27. ¹³C-NMR spectrum of compound 3i.

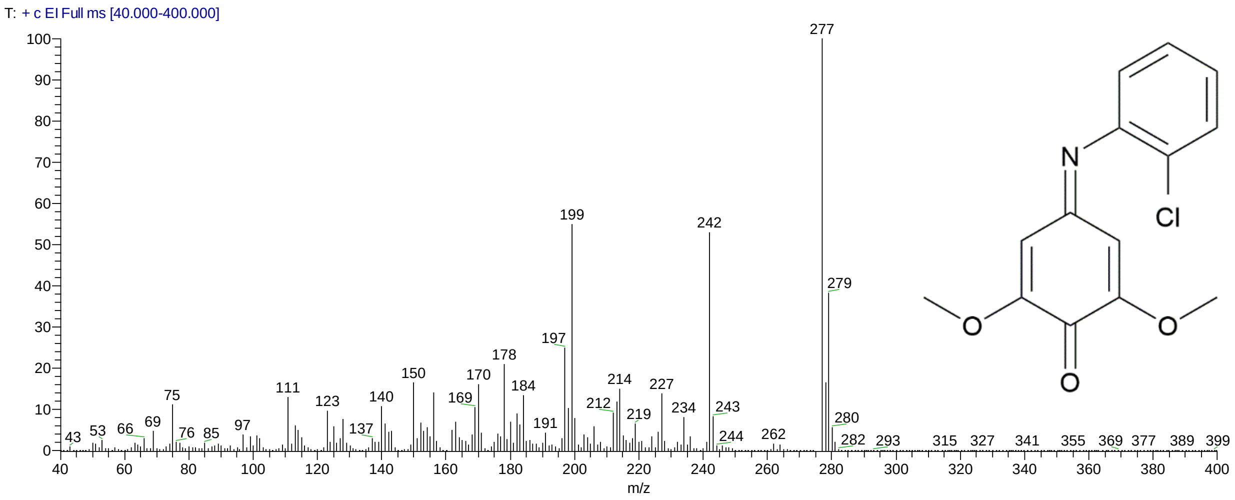

Figure S28. Mass spectrum of compound 3j.

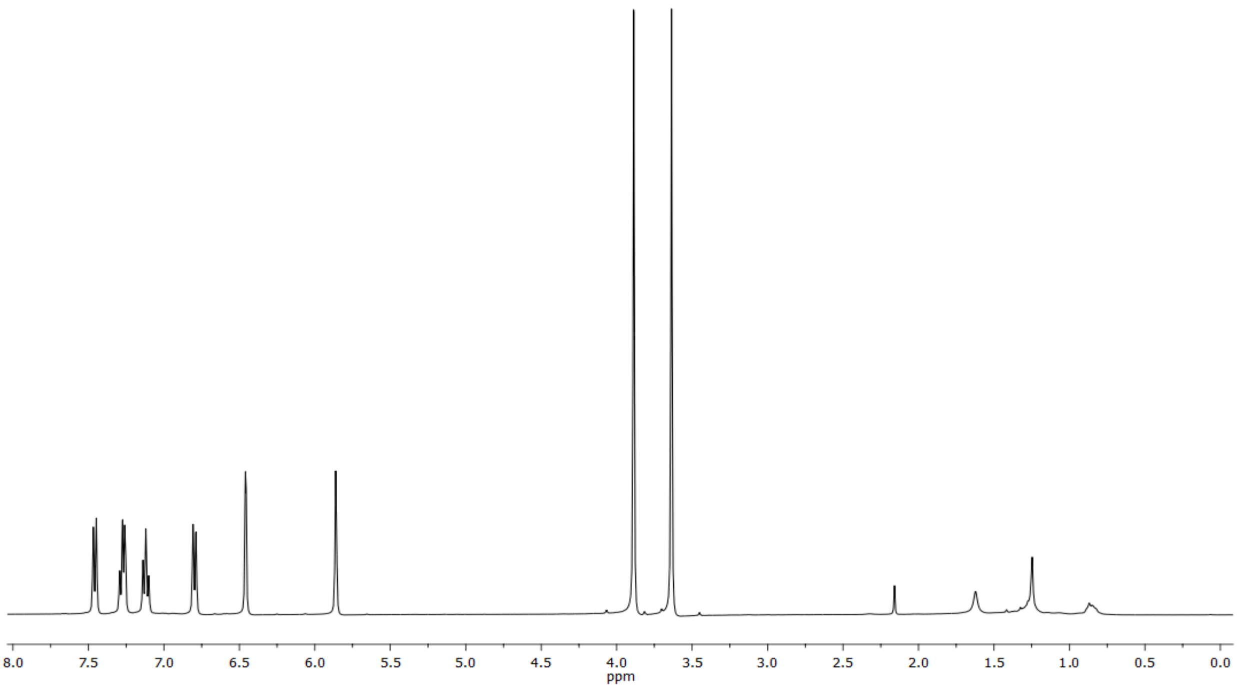

Figure S29. ^{1}H-NMR spectrum of compound 3j.

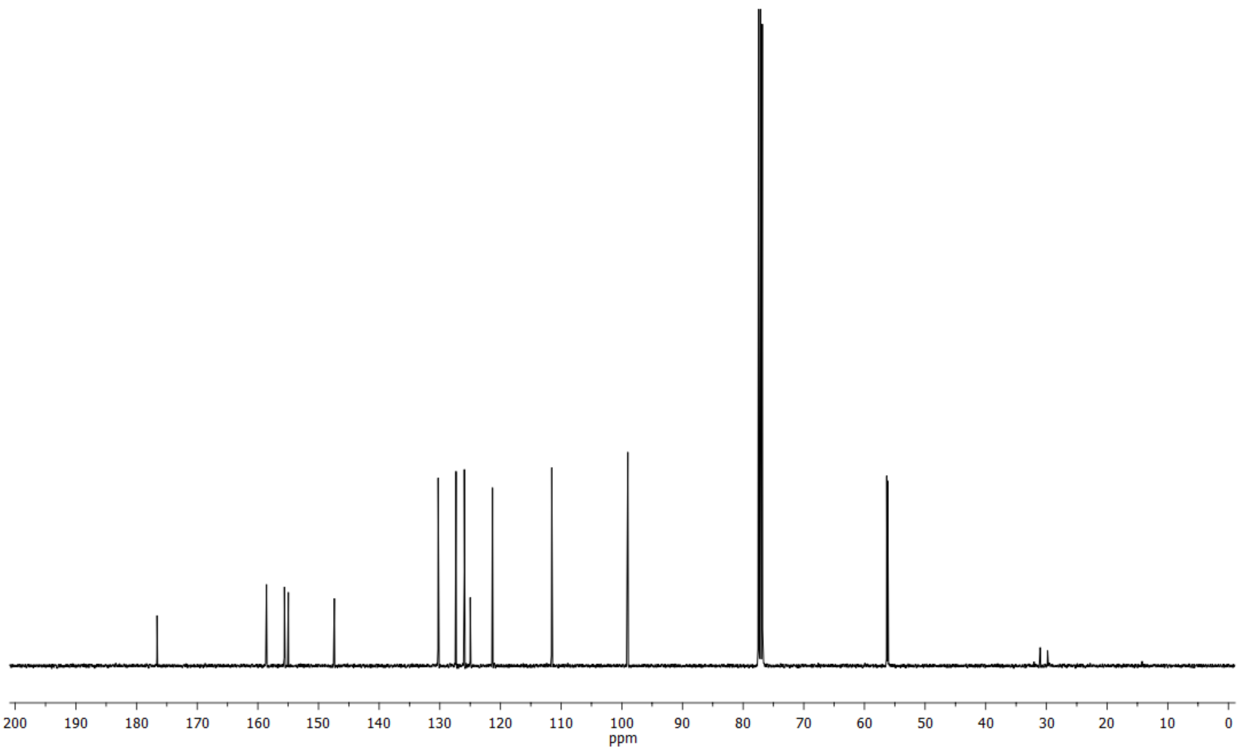

Figure S30. ^{13}C-NMR spectrum of compound 3j.

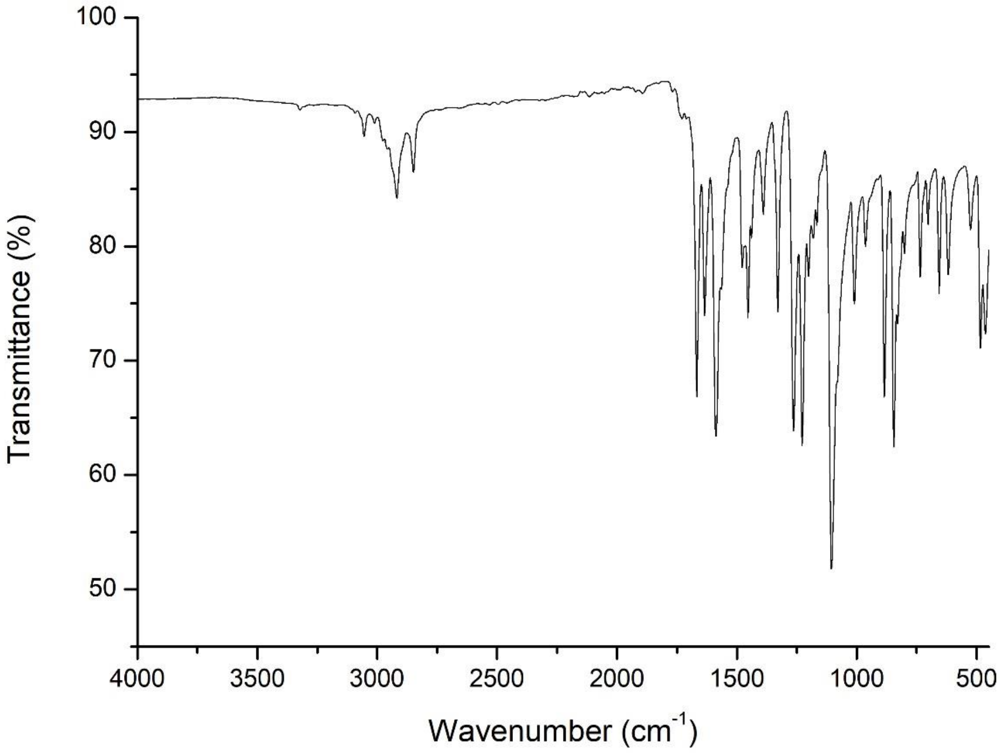

Figure S31. IR spectrum of compound 3a .

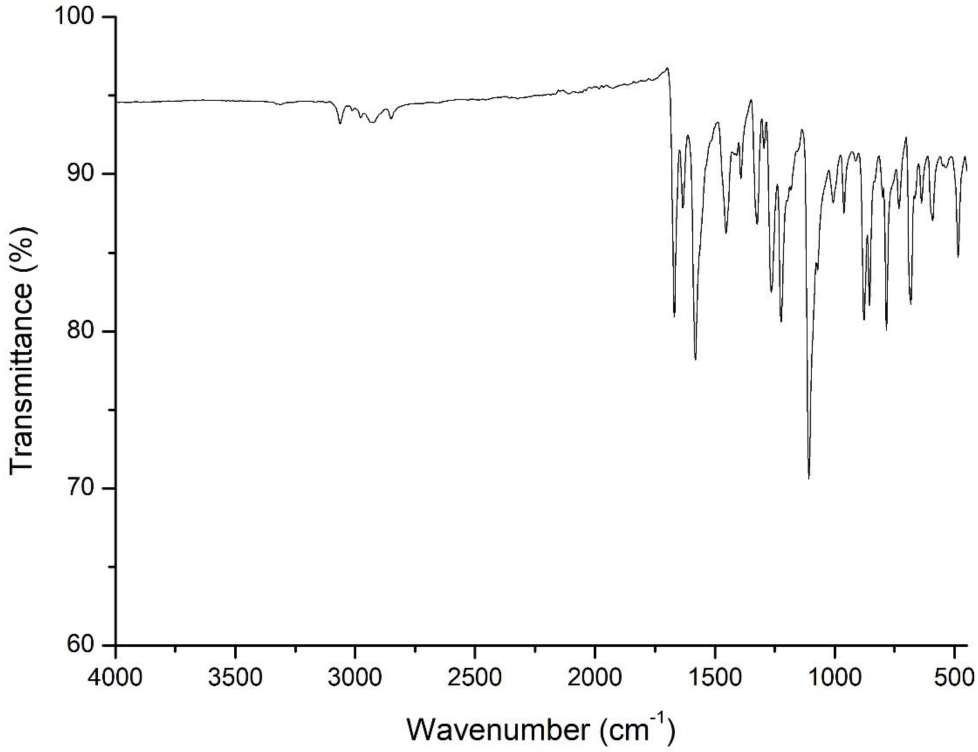

Figure S32. IR spectrum of compound 3b.

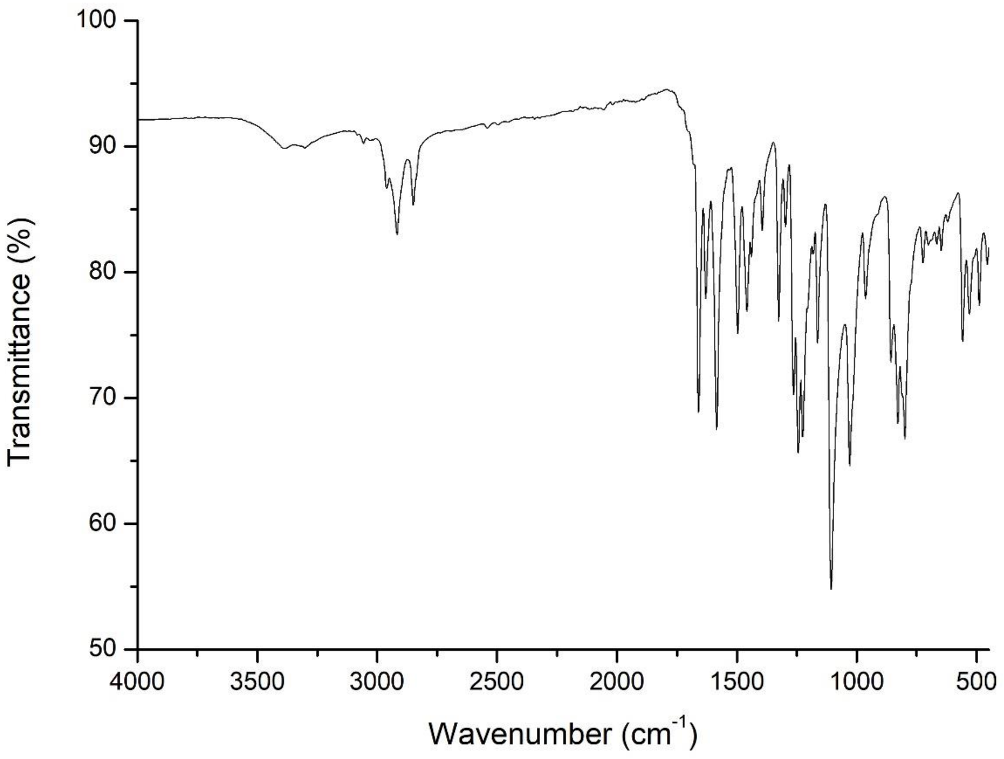

Figure S33. IR spectrum of compound 3c.

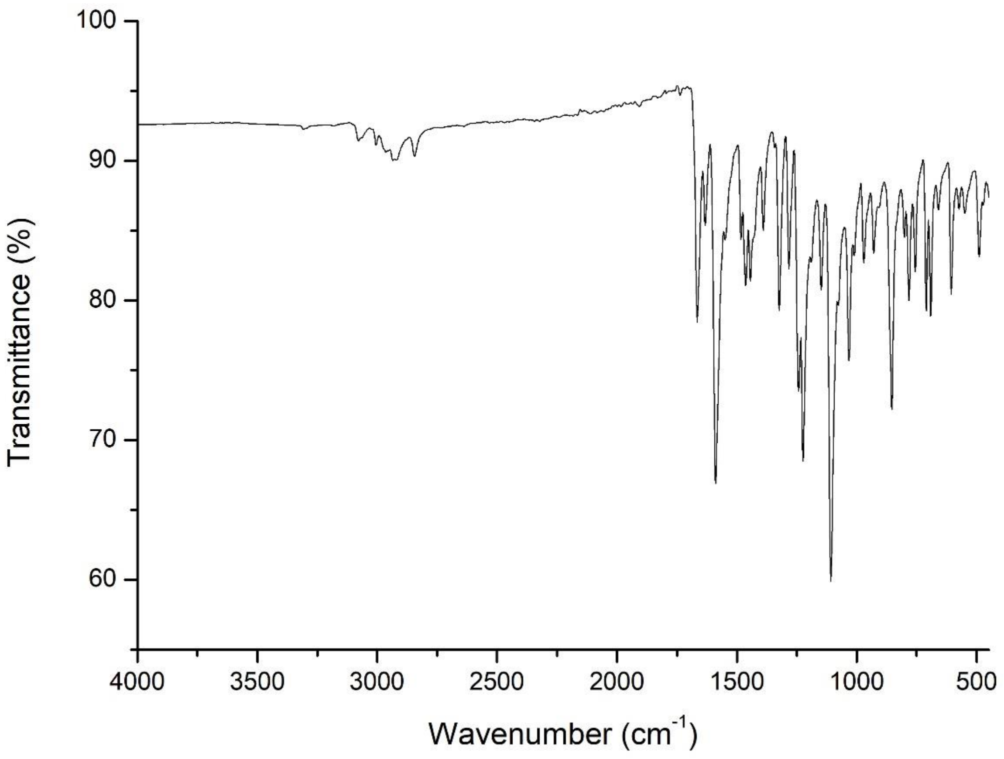

Figure S34. IR spectrum of compound 3d.

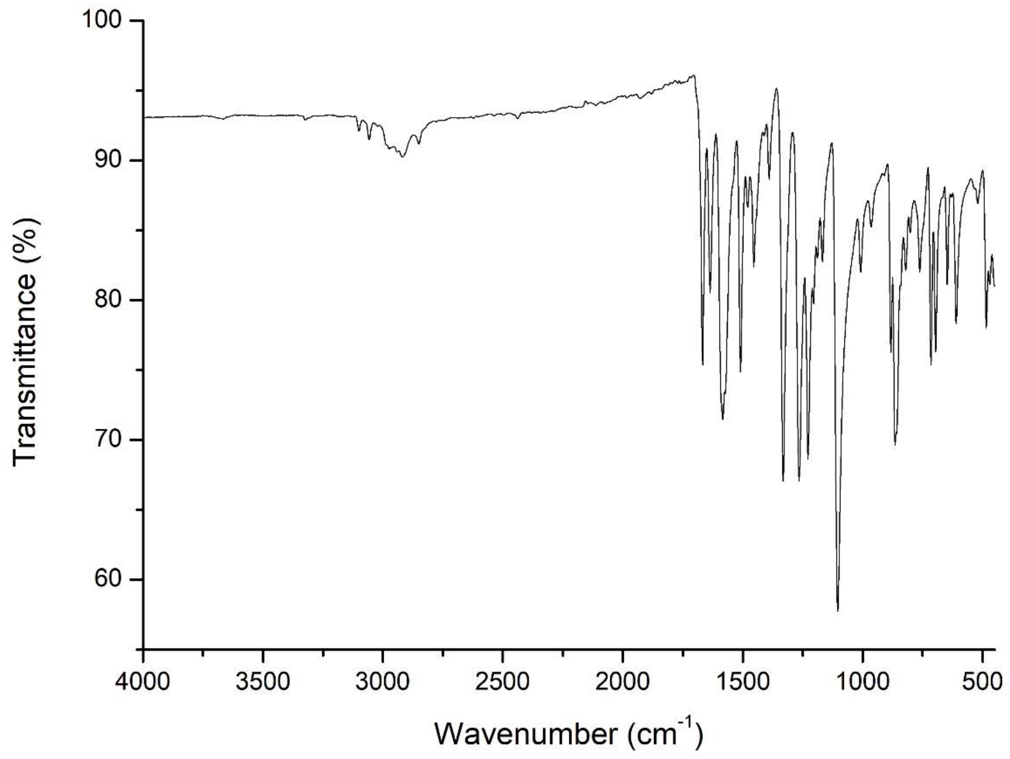

Figure S35. IR spectrum of compound 3e.

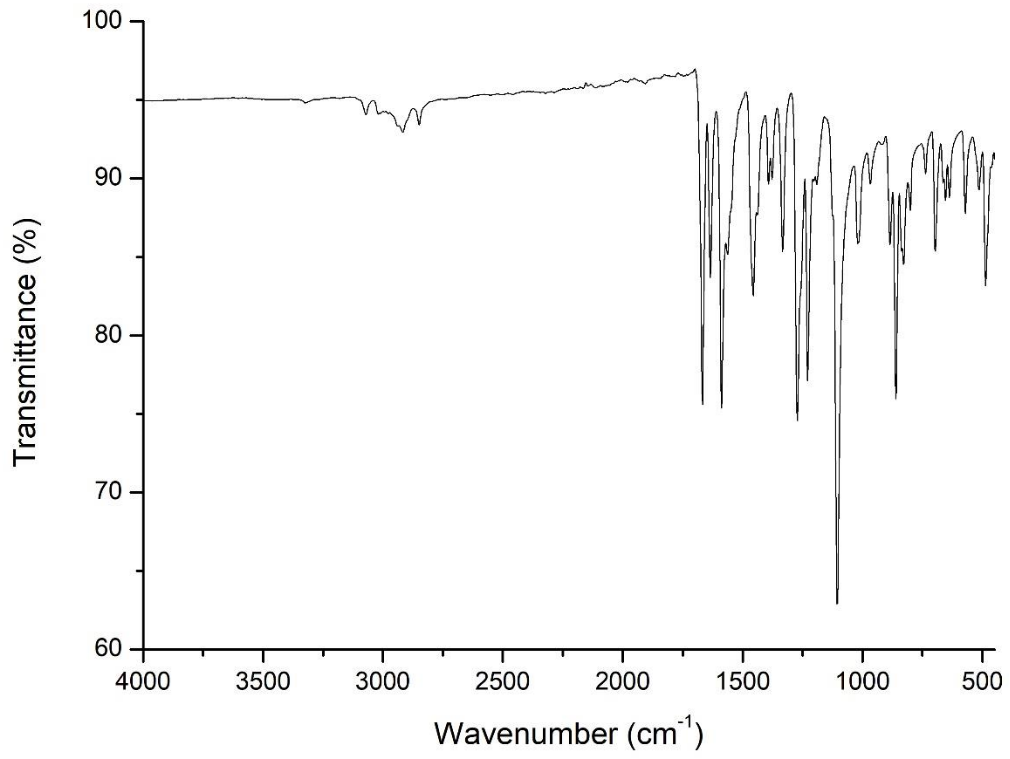

Figure S36. IR spectrum of compound 3f.

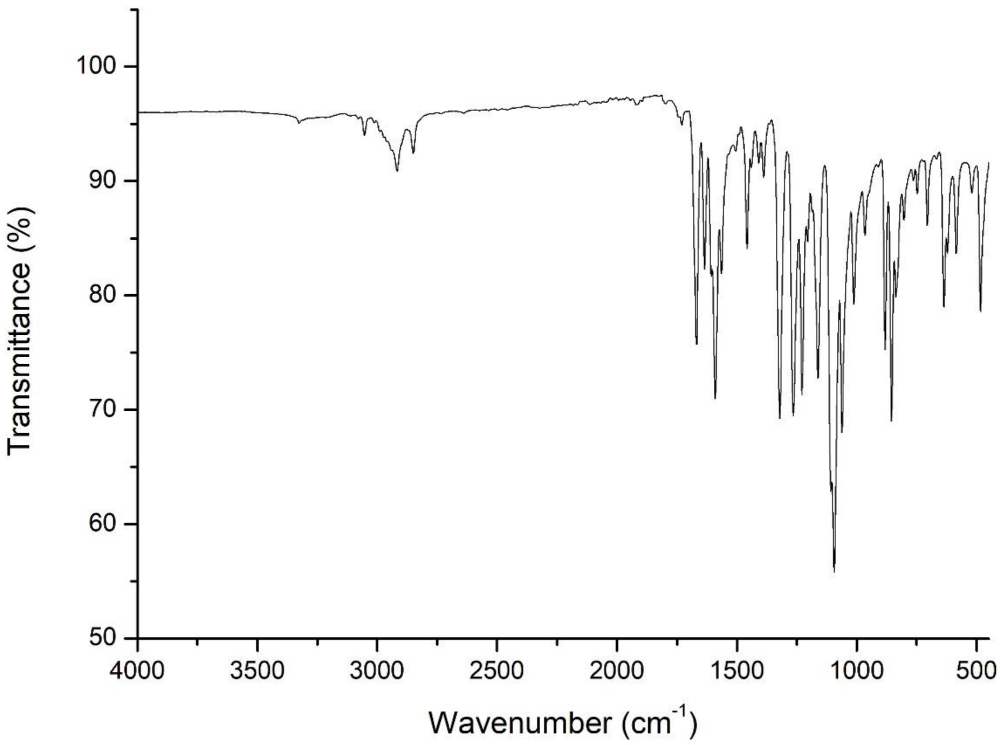

Figure S37. IR spectrum of compound 3g.

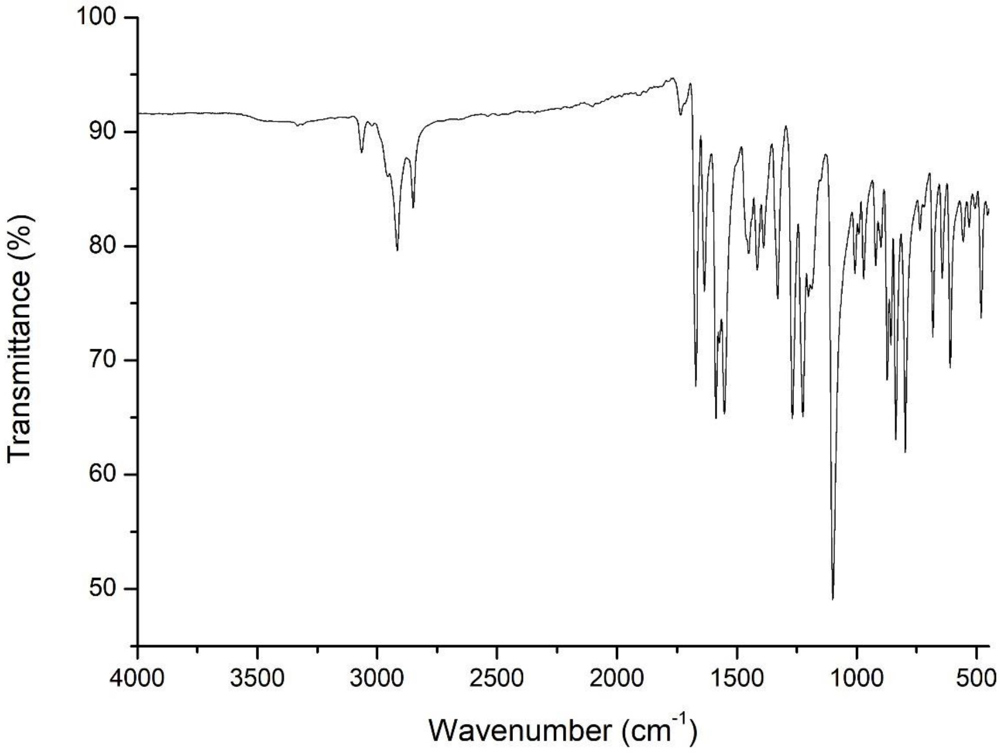

Figure S38. IR spectrum of compound 3h.

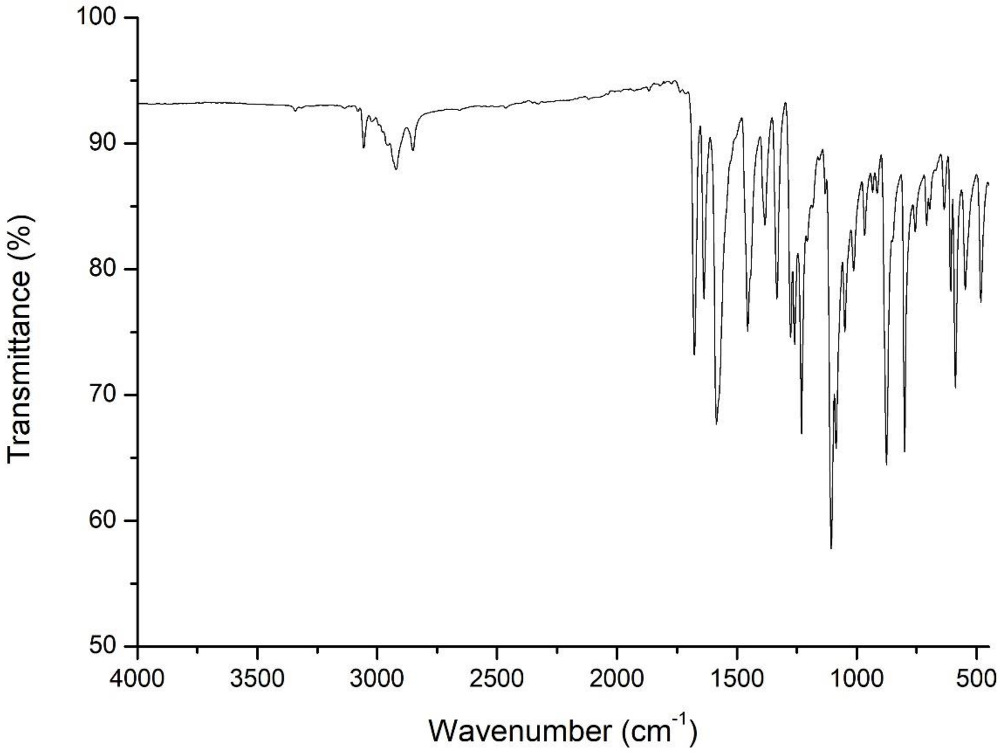

Figure S39. IR spectrum of compound 3i.

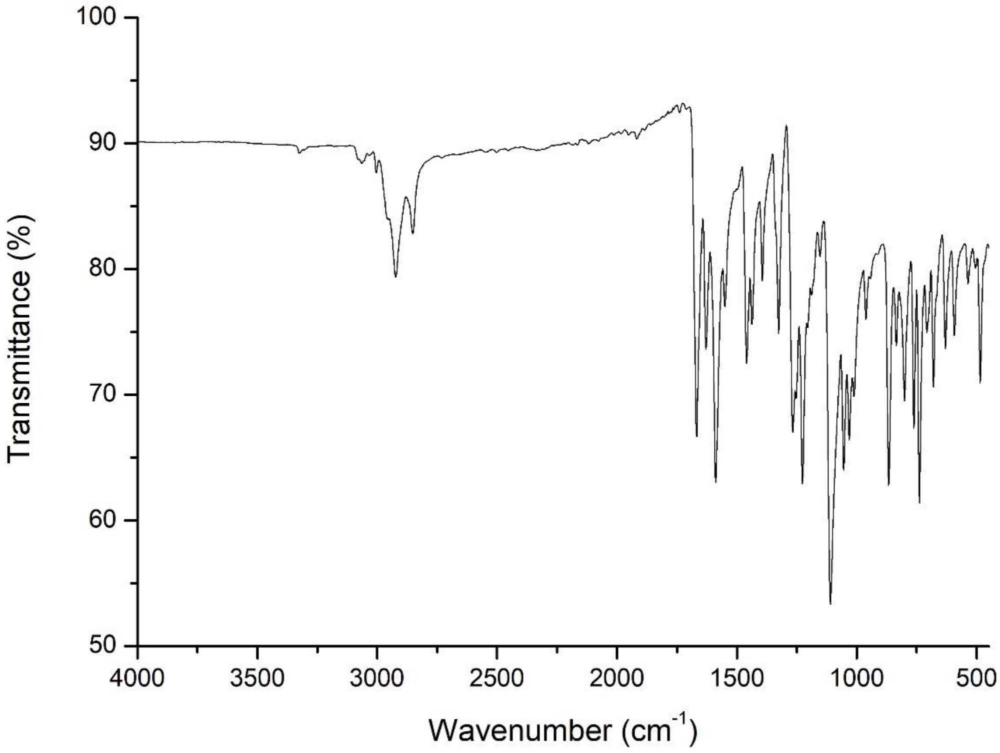

Figure S40. IR spectrum of compound 3j.

Table S1. Retention index of compounds 3a-j

N°	Compound	Retention Index	Molecular Formula

1	3a	2366	$C_{14}H_{12}O_3NCl$
2	3b	2340	$C_{14}H_{12}O_3NCl$
3	3c	2451	$C_{15}H_{15}O_4N$
4	3d	2403	$C_{15}H_{15}O_4N$
5	3e	2690	$C_{14}H_{12}O_5N_2$
6	3f	2556	$C_{14}H_{11}O_3NCl_2$
7	3g	2084	$C_{15}H_{12}O_3NF_3$
8	3h	2472	$C_{14}H_{11}O_3NCl_2$
9	3i	2449	$C_{14}H_{11}O_3NCl_2$
10	3j	2300	$C_{14}H_{12}O_3NCl$
